# Proteomic signatures during pregnancy: an exploratory cohort study of long-term health indicators

**DOI:** 10.1016/j.ebiom.2026.106491

**Published:** 2026-09-22

**Authors:** Lucy A. Bartho, Susan P. Walker, Teresa MacDonald, Danica Idzes, Ping Cannon, Tuong-Vi Nguyen, Gordon Smith, D. Stephen Charnock-Jones, Stephen Tong, Tu'uhevaha J. Kaitu'u-Lino

**Affiliations:** a Translational Obstetrics Group, The Department of Obstetrics and Gynaecology, Mercy Hospital for Women, University of Melbourne, 163 Studley Road, Heidelberg, 3084, Victoria, Australia; b Mercy Perinatal, Mercy Hospital for Women, Victoria, Australia; c Department of Obstetrics and Gynaecology, NIHR Cambridge Biomedical Research Centre, University of Cambridge, Cambridge, UK; d Loke Centre for Trophoblast Research (LCTR), Department of Physiology, Development and Neuroscience, University of Cambridge, Cambridge, UK

**Keywords:** SomaSignal test, Long-term, Preeclampsia, Fetal growth restriction, Biomarker, Proteomics

## Abstract

**Background:**

Pregnancies complicated by fetal growth restriction (FGR) and preeclampsia are associated with increased long-term chronic disease risk. A blood test could possibly identify those at risk, opening the door to early interventions soon after delivery. Prior work in non-pregnant people have identified protein signatures associated with 18 different health parameters, called SomaSignal Tests.

**Methods:**

This study applied SomaSignal tests to plasma samples collected in pregnancy from two independent cohorts to assess the 18 health parameters in those who later developed FGR and preeclampsia. SomaScan Assay measured ∼7000 modified aptamers in plasma from two independent cohorts. Plasma samples (Melbourne, Australia) were collected at 36 weeks' gestation from women who later delivered an FGR infant (<3rd birthweight centile, n = 118), preeclampsia at term (n = 92) or did not develop either condition (n = 177). The second cohort (Cambridge, UK) were plasma samples collected at 36 weeks’ gestation from women who later delivered an FGR infant (n = 80), and those who did not (n = 172).

**Findings:**

In the Australian cohort, women who later delivered growth-restricted infants had composite protein signatures associated with reduced cardiac function (p = 0.001), increased cardiovascular events (p = 7.6 × 10^−6^), mid-life dementia (p = 1.5 × 10^−9^), and higher visceral fat (p = 4.06 × 10^−6^), compared to pregnancies without complications. Findings were largely consistent in both Australian and UK sample sets. Those who developed preeclampsia had protein signatures signifying cardiovascular (p = 5.54 × 10^−6^), hepatic (p = 0.001), and renal (p = 0.0007) disease risks, compared to pregnancies without complications.

**Interpretation:**

Since signatures were more pronounced in pregnancies that were later diagnosed with FGR and preeclampsia, underlying physiological changes may have been present before diagnosis. These changes result in protein signatures concordant with those associated with poor long-term health outcomes in non-pregnant populations. These associations require validation in large, longitudinal cohorts investigating pregnancy, with long-term maternal follow-up.

**Funding:**

Stillbirth CRE, Mercy Hospital Foundation and National Health and Medical Research Council, Royal Australian and New Zealand College of Obstetricians and Gynaecologists, Taylor Hammond Scholarship, National Health and Medical Research Council Fellowships and Australian Research Council Future Fellowships.

**Research in context**

**Evidence before this study**

Pregnancy complications such as fetal growth restriction (FGR) and preeclampsia are strongly associated with maternal adverse long-term outcomes such as cardiovascular, metabolic and neurodegenerative disease. However, no blood tests can provide early identification of women at risk of these long-term diseases or identify women at risk of developing pregnancy complications.

**Added value of this study**

This study assessed SomaSignal health indicator signatures in pregnancy before diagnosis of fetal growth restriction, or preeclampsia. Using aptamer-based proteomics, 7000 plasma proteins were measured at 36 weeks’ gestation, and 18 previously trained SomaSignal algorithms were applied. These SomaSignal Tests reflect diverse chronic disease pathways, including cardiovascular, hepatic, renal, and neurodegenerative outcomes. We demonstrate that women who delivered a growth-restricted infant exhibit plasma protein profiles associated with elevated long-term risk for cardiovascular disease, dementia, and metabolic dysfunction. Importantly, these findings were replicated across two cohorts. Similarly, women who later developed preeclampsia had signatures associated with increased risk of cardiovascular, hepatic, and renal disease.

**Implications of all the available evidence**

We have identified proteomic signatures in women during pregnancy; before diagnosis of fetal growth restriction or preeclampsia with shared biological pathways as individuals with long-term chronic disease. These data provide insights into the possibility of utilising a test during pregnancy to provide information about future health risks. However further validation in larger longitudinal cohorts is required to confirm this.

## Introduction

Pregnancy serves as the ultimate physiological stress test.^1^^,^^2^ To support fetal growth, women undergo critical anatomical and functional adaptations.^3^ When these adaptations are inadequate, pregnancy complications such as fetal growth restriction (FGR) and preeclampsia can occur. These complications may give rise to other health complications that only become clinically apparent years later. Epidemiological studies have identified associations between pregnancy complications and increased risk of long-term chronic diseases.^4^, ^5^, ^6^ These include cardiovascular disease,^7^, ^8^, ^9^ impaired glucose tolerance,^8^ renal dysfunction,^10^ dementia^11^, ^12^, ^13^ and others. An accurate test that could be added to routine blood tests during pregnancy to identify women at future risk could open the door to early counselling, personalised care, and early interventions soon after delivery^14^^,^^15^ to reduce chronic disease risk.

We utilised SomaLogic technology^16^–a high-throughput proteomics platform that measures over 7000 plasma proteins. We then applied SomaSignal tests to our protein data.^16^, ^17^, ^18^, ^19^ These are machine learning (ML) algorithms, trained on large, non-pregnant population datasets to detect protein signatures associated with chronic disease risk across 18 health indicators (available at the time of measurement). These include: cardiovascular events,^19^ cardiorespiratory fitness, mid-life dementia,^20^ metabolic, renal and hepatic dysfunction,^21^ body composition (body fat and lean mass), and alcohol-related impacts. While the SomaSignal tests have been validated and published extensively in the general population,^19^, ^20^, ^21^ data applying these to pregnancy has not been published.

We applied the SomaSignal tests to proteomic data from maternal plasma collected at 36 weeks' gestation from two independent, prospective cohorts. This was an exploratory analysis where our aim was to determine whether we can identify proteomic signatures concordant with those previously associated with future chronic disease risk (in non-pregnant populations^20^). To generate evidence that these tests may possibly predict future disease risk, we compared SomaSignal tests in plasma collected at 36 weeks’ gestation, prior to the detection of term FGR (<3rd birthweight centile neonate), or preeclampsia to unaffected pregnancies. As these conditions are linked with future chronic diseases, we hypothesise SomaSignal Tests will be abnormal in women who later develop these pregnancy conditions compared with samples from women who do not develop these conditions.

## Methods

### Discovery cohort

The fetal longitudinal assessment of growth (FLAG)^22^^,^^23^ and biomarker and ultrasound measures for preventable stillbirth (BUMPS)^24^^,^^25^ studies are large, prospective studies conducted at the Mercy Hospital for Women, Melbourne, Australia. Both studies were designed to identify biomarkers for pregnancy pathologies: FGR and preeclampsia.

FLAG is a large, prospective study commenced from January 2015 until December 2017. Approximately 2000 plasma samples were collected at 36 weeks’ gestation (35^+0^–37^+0^)^22^^,^^23^ and pregnancy records assessed to determine outcomes after birth. As this study was an exploratory analysis, we deliberately selected all samples with pregnancy condition (FGR and/or preeclampsia) and a random selection of pregnancies that did not have complications. This study was part of a larger study collected from the FLAG (2000 participants) and BUMPS 2000 cohorts (commenced from February 2019 until October 2025). These samples are representative of pregnant women in Melbourne, Victoria. The study includes nulliparous women with singleton pregnancies. We included, 118 who delivered an FGR infant (<3rd birthweight percentile), 92 who developed preeclampsia (after sample collection) and 177 randomly selected participants using standard R indexing approach in R studio. Participants who did not develop either condition (FGR, or preeclampsia), as the controls. We included all participants who were diagnosed with both FGR and preeclampsia and excluded those carrying more than one baby. Controls were not matched to cases based on maternal characteristics.

Birthweight centile was defined by GROW 2022 (Australia) birthweight centile calculator^26^ (unadjusted for ethnicity), accounting for fetal sex (determined by health records) and gestation specific and adjusted for maternal height, weight and parity, where <3rd birthweight centile was defined as fetal growth restriction.^27^^,^^28^ Preeclampsia was diagnosed according to the ACOG guidelines, following the development of preeclampsia after 37 weeks’ gestation.^29^ For this cohort, routine clinical care at the 36-week appointment did not identify any signs or symptoms of preeclampsia, based on clinical blood pressure measurements. There was also no indication of FGR. Whole blood was collected in 9 mL ethylenediaminetetraacetic acid (EDTA) tubes, tubes centrifuged at 1000×*g* for 10 min, and plasma was collected, aliquoted, and stored at −80 °C until sample analysis.

### Replication cohort

The pregnancy outcome prediction (POP) study is a large, prospective cohort study collected at the Rosie Hospital, Cambridge, UK, conducted between 2008 and 2012. The study includes nulliparous women with singleton pregnancies. The POP study has been previously described.^30^^,^^31^

Maternal plasma was collected at 36 weeks’ gestation (35^+0^–37^+0^) and pregnancy outcomes determined at birth. For this cohort, routine clinical care at the 36-week appointment did not identify any signs or symptoms of preeclampsia, based on clinical blood pressure measurements. There was also no indication of FGR.^30^

Birthweight centile was determined using customised birth weight centiles which account for fetal sex and gestation specific and adjusted for maternal height, weight and parity.^32^ The transformation of data was performed with the Scottish GROW centiles bulk calculator.^33^ A different GROW chart was applied compared to the Australia samples, as the charts are population specific and validated for each region. These charts were designed to adjust for population demographics. In this study, <3rd birthweight centile was defined as fetal growth restriction.^34^ Samples from the POP cohort includes 80 who delivered an infant with FGR (<3rd birthweight percentile), and 172 who did not develop either condition (FGR, or preeclampsia) as controls. The controls were randomly selected using standard R indexing approach in R studio (version 2024.12.1 + 563), and controls were not matched to cases based on maternal characteristics.

### Ethics

Ethical approval for FLAG was obtained from the Mercy Health and Human Research Ethics Committee (Ethics Approval Number: R14/12). BUMPS is a prospective study to collect plasma at 36 weeks’ gestation (35^+0^–37^+0^),^35^^,^^36^ with pregnancy outcomes similarly determined at birth. Ethical approval for BUMPS was obtained from the Mercy Health and Human Research Ethics Committee (Approval number: 2019–012). For POP cohort, ethical approval was obtained from the Cambridgeshire 2 Research Ethics Committee (reference number 07/H0308/163). All participants in this study provided written informed consent. This study adhered to the Declaration of Helsinki.

### Proteomic platform

The SomaScan Assay and its modified aptamer binding reagents have been previously described.^37^^,^^38^ The protein abundance was measured using the 7 k SomaScan assay, version 4.1 by SomaLogic, Inc (Boulder, CO) The platform uses ∼7000 individual DNA-based slow off-rate modified aptamers (SOMAmer reagents) to bind target proteins in diluted plasma samples. To ensure specificity, the assay includes multiple steps to remove non-specific binding.^39^ Finally, the DNA-based reagents are quantified through hybridisation on an Agilent array (Agilent Technologies, Inc., Santa Clara, CA). Sample processing of plasma from each cohort was matched before shipment to SomaLogic for proteomic analysis. To minimise technical variation within the proteomic analysis, sample processing included multiple internal standards, and strict quality control procedures were employed. Samples were run on a 96-well plate, and each plate contained 11 external control wells, including: five calibrator samples, three quality control samples, and three buffer blanks, which were used to normalise raw fluorescence signals and monitor assay performance across plates. Three fixed serial dilutions (0.05%, 0.5%, and 20%) were used for optimisation of dynamic range (low and high abundance) of aptamers. Standardisation of raw data using external quality controls were used to correct for intra- and inter-plate variation. Quality control metrics (e.g. cross-plate QC flags, and proportion below the limit of detection) were used to identify and poorly performing aptamers. Limits of detection were defined using buffer controls.^39^ These steps were used to ensure high technical reproducibility and minimise variation between assay runs.

### SomaSignal tests

The SomaSignal tests applied to the discovery (BUMPS and FLAG cohorts) and replication (POPS cohort) datasets were previously developed for research purposes in non-pregnant populations.^17^^,^^19^^,^^20^^,^^29^ SomaSignal Tests were generated using pre-trained composite algorithms derived from large-scale proteomics datasets.^17^^,^^19^^,^^20^^,^^29^ They were not originally designed or calibrated to predict long-term disease outcomes in pregnancy. Each SomaSignal Test represents an aggregate proteomic signature relevant to each physiological status. Using our proteomic data generated from the SomaScan platform, we applied the SomaSignal Tests for 18 health indicators: Primary and secondary cardiovascular event risk, cardiopulmonary fitness, mid-life dementia risk (within 20 years), heart failure prognosis (based off proteomic profiles from patients diagnosed with stable heart failure), including: heart failure with preserved ejection fraction (within 6 months), heart failure with preserved ejection fraction (within 12 months), heart failure with reduced ejection fraction (within 6 months), and heart failure with reduced ejection fraction (within 12 months) visceral fat, glucose tolerance, kidney prognosis, body fat percentage, liver fat, non-alcoholic steatohepatitis (NASH) ballooning, non-alcoholic steatohepatitis fibrosis, resting energy rate, lean body mass and alcohol impact. Previous analyses that developed the SomaSignal Tests derived risk bins and survival models using large datasets where longitudinal data was collected, and disease outcomes were known. For each disease domain, either an accelerated failure time-Weibull model with elastic net regularisation, accelerated failure time (AFT)-Weibull model with Lasso regularisation, regression model with elastic net regularisation, AFT-Weibull model, logistic regression model with elastic net regularisation, elastic net regression model, elastic net linear regression model, elastic net logistic regression or elastic net logistic regression modelling approach was employed (see Supplementary Table S1). SomaSignal outputs are the result of composite algorithm-derived measures, rather than single protein concentrations. In the present study, risk scores and bins derived in non-pregnant participants^17^^,^^19^^,^^20^^,^^29^ were applied to the protein outputs we obtained from our samples collected during pregnancy. This current study was therefore an exploratory analysis, and conclusions require validation in sample sets that include long-term maternal outcomes. The primary aim was to compare median SomaSignal-derived health scores between women who subsequently developed pregnancy complications and women with uncomplicated pregnancies within the Australian FLAG/BUMPS cohort. Our secondary aim was to determine whether similar patterns would be replicated in the independent POP cohort.

### Statistics

Maternal characteristics were compared between pregnant women who later developed FGR and/or preeclampsia and those who did not. Characteristics were compared using Mann–Whitney U test for continuous data and Fisher's exact test for categorical data was used.

For each individual test, the pregnancy outcome (fetal growth restriction, preeclampsia or control) is the independent variable, and the SomaSignal test is the dependent variable. Each SomaSignal test represents a predefined clinical domain, evaluated in independent cohorts and not as multiple comparisons of a single endpoint. From a SomaSignal test you get a single continuous outcome for each chronic condition. High scores indicate a greater risk of developing the chronic disease in the future. Thirteen of the 18 tests can be considered categorically. For the categorical tests (13/18), SomaLogic provided predefined categorical risk groups (bins) based on the reference population data. These bins were not generated by our team. Participants were assigned to categories according to their continuous SomaSignal score. These enable different risk category, based on each different test (e.g. Dementia risk categories: low; medium-low; medium–high; high). All continuous SomaSignal data generated was tested for normality using Shapiro–Wilk test. If it was considered not normally distributed, a Mann–Whitney (non-parametric) test was used to compare the mean/median between those with pregnancy complications and those without. The data is presented graphically as median interquartile range [IQR]. Categorical SomaSignal data is presented as proportions. The proportion in the high-risk category for those with pregnancy complications and those without was compared using Fisher's exact test.

Given the pregnancy complication and no pregnancy complication groups differed on key demographic variables, these were adjusted for in our quantile regression (τ = 0.5) model to estimate median differences between groups. R studio version 2024.12.1 + 563, “quantreg” package (version 6.1) was used for the analysis. Unadjusted and adjusted median differences with 95% confidence intervals were presented in Supplementary Table S3 (Discovery cohort–FLAG and BUMPS; FGR), Supplementary Table S5 (Replication cohort–POP; FGR) and Supplementary Table S6 (Discovery cohort–FLAG and BUMPS; preeclampsia). The adjusted median differences were adjusted for maternal age, maternal BMI, smoking status, and gestational diabetes status.

Results in text were reported as unadjusted p values, and 95% confidence intervals. p < 0.05 was considered significant. Results were interpreted cautiously, with emphasis placed on findings replicated across independent cohorts. All results were interpreted as exploratory and require independent validation. All analysis was performed using GraphPad Prism 9.3.1 (GraphPad Software, LLC) or exact p values were generated using R Studio version 2024.12.1 + 563.

### Role of funders

The funders had no role in the design, data collection, data analyses, interpretation, or writing of this study.

## Results

The primary aim was to compare median SomaSignal-derived health scores between women who subsequently developed pregnancy complications and women with uncomplicated pregnancies within the Australian FLAG/BUMPS cohort. The cohort includes 118 women who delivered infants with fetal growth restriction, 92 who later developed preeclampsia, and 177 pregnancies unaffected by either condition (control). We used plasma samples collected at 36 weeks’ gestation. Clinical characteristics are shown in Supplementary Table S2. Those who delivered with FGR, were likely to be older (maternal age, p = 0.03, Mann Whitney U test), have reduced onset of labour (p = 0.03, Chi-square test) and earlier gestation at delivery (p < 0.0001, Mann Whitney U test, Supplementary Table S2). The groups had no clinically important differences regarding maternal body mass index (BMI), parity, smoking status, gestational diabetes mellitus status, mode of birth and sex of the baby (Supplementary Table S2). Those who developed preeclampsia were likely to have reduced parity (p = 0.0007, Chi-square test), type of labour (p = 0.01, Chi-square test), increased emergency caesarean section (p = 0.009, Chi-square test), earlier gestation at delivery (p = 0.0002, Mann Whitney U test), reduced birthweight (p = 0.0004, Mann Whitney U test), and birthweight centile (p = 0.03, Mann Whitney U test, Supplementary Table S2). Maternal age, maternal BMI, smoking status, GDM status, and fetal sex had similar risk profiles between groups (Supplementary Table S2).

Compared to the control group, those who later delivered a growth restricted infant had SomaSignal Tests associated with 1) increased primary cardiovascular risk (median 0.6 control vs. 0.8 FGR, p = 7.6 × 10^−6^; Mann Whitney U test, Fig. 1a; unadjusted and adjusted median differences presented in Supplementary Table S3), with a 4.43-fold increase in patients classified as medium-low risk (10.2% FGR vs. 2.3% control, p = 0.006, Fisher's exact test, Table 1), 2) reduced cardiorespiratory fitness (VO_2,_ median 21.3 control vs. 20.8 FGR; p = 0.0098, Mann Whitney U test, Fig. 1c; unadjusted and adjusted median differences presented in Supplementary Table S3), 3) increased risk of heart failure with reduced ejection fraction (HFrEF) within 6 months (based on proteomic profiles from patients diagnosed with stable heart failure, median 1.2 control vs. 1.5 FGR; p = 0.001, Mann Whitney U test, Fig. 1d; unadjusted and adjusted median differences presented in Supplementary Table S3) and 12 months (median 2.1 control vs. 2.6 FGR; p = 0.001, Mann Whitney U test, Fig. 1e; unadjusted and adjusted median differences presented in Supplementary Table S3) and 4) increased risk of mid-life dementia (median 0.167 control vs. 0.279 FGR; p = 1.5 × 10^−9^, Mann Whitney U test, Fig. 1h; unadjusted and adjusted median differences presented in Supplementary Table S3), with a 2.12-fold increase in patients classified as high risk (61% FGR vs. 28.8% control, p < 0.0001, Fisher's exact test, Table 1). Residual cardiovascular risk (Fig. 1b), heart failure with preserved ejection fraction (HFpEF) within 6 months (Fig. 1f) and 12 months (Fig. 1g) had similar risk profiles between groups.

**Fig. 1:**
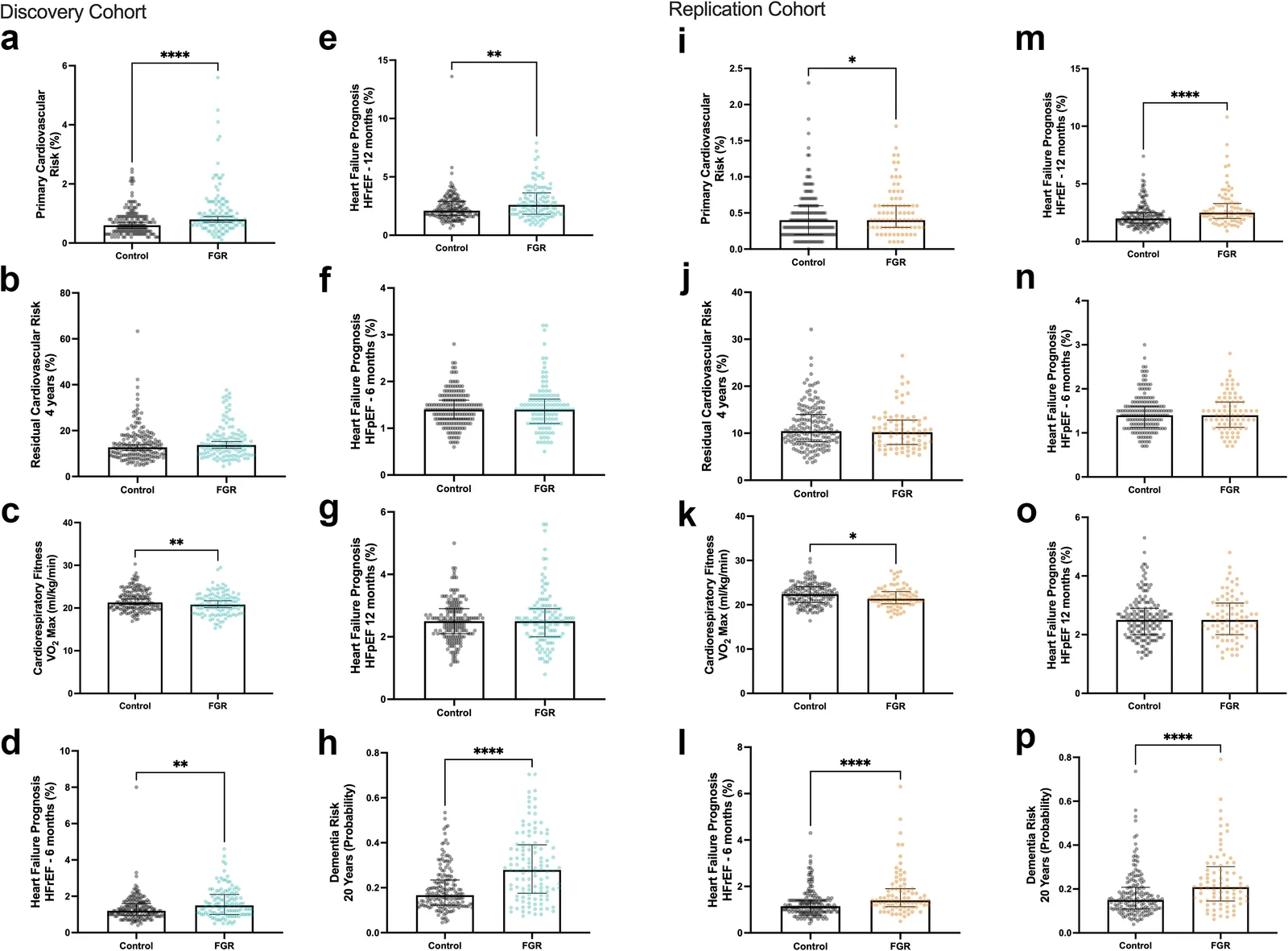
Bar graphs with median interquartile range (IQR) of SomaSignal Tests in two large cohorts where plasma was collected from pregnant women at 36 weeks' gestation, who subsequently delivered with fetal growth restriction (FGR), with birthweight <3rd centile, or not >3rd birthweight centile (control) (**a–h**, Discovery cohort, **i–p**, Replication cohort). Risk of a primary cardiovascular event (**a**), residual cardiovascular risk (**b**), cardiorespiratory fitness (peak exercise capacity, VO_2_, **c**), heart failure with reduced ejection fraction (HFrEF) within 6 months (**d**), and 12 months (**e**), heart failure with preserved ejection fraction (HFpEF) within 6 months (**f**), and 12 months (**g**), risk of mid-life dementia (**h**) in the discovery cohort, collected at a hospital in Melbourne, Australia (Control, n = 177, FGR, n = 118). Risk of primary cardiovascular event (**i**), residual cardiovascular risk, (**j**), cardiorespiratory fitness (**k**), heart failure with reduced ejection fraction (HFrEF) within 6 months (**l**), and 12 months (**m**), heart failure with preserved ejection fraction (HFpEF) within 6 months (**n**), and 12 months (**o**), risk of mid-life dementia (**p**) in the replication cohort, collected at a hospital in Cambridge, UK (Control, n = 172, FGR, n = 80). Groups were compared using Mann–Whitney U tests with median ± IQR. ∗p < 0.05, ∗∗p < 0.01, ∗∗∗p < 0.001, ∗∗∗∗p < 0.0001.

**Table 1:** SomaSignal test survival model risk bins.

SomaSignal test	Risk bins for survival models	Control (n = 177) (%)	Fetal growth restriction (n = 118) (%)	p-value	Preeclampsia (n = 92) (%)	p-value
Primary cardiovascular risk	Low	173 (97.7%)	106 (89.8%)	0.006	83 (90.2%)	0.01
	Medium-Low	4 (2.3%)	12 (10.2%)		9 (9.8%)	
	Medium-High	0 (0%)	0 (0%)		0 (0%)	
	High	0 (0%)	0 (0%)		0 (0%)	
Residual cardiovascular risk	Low	21 (11.8%)	9 (7.6%)	0.45	2 (2.2%)	0.001
	Medium -Low	141 (79.7%)	96 (81.4%)		71 (77.2%)	
	Medium-High	14 (7.9%)	13 (11%)		18 (19.6%)	
	High	1 (0.6%)	0 (0%)		1 (1%)	
Cardiorespiratory fitness	Low	172 (97.2%)	116 (98.3%)	0.71	91 (98.9%)	0.67
	Fair	5 (2.8%)	2 (1.7%)		1 (1.1%)	
	Good	0 (0%)	0 (0%)		0 (0%)	
	Excellent	0 (0%)	0 (0%)		0 (0%)	
Dementia	Low	6 (3.4%)	0 (0%)	<0.0001	0 (0%)	<0.0001
	Medium-Low	64 (36.2%)	23 (19.5%)		13 (14.1%)	
	Medium-High	56 (31.6%)	23 (19.5%)		26 (28.3%)	
	High	51 (28.8%)	72 (61%)		53 (57.6%)	
Visceral fat	20th centile	2 (1.1%)	0 (0%)	0.0003	0 (0%)	<0.0001
	60th centile	61 (34.5%)	21 (17.8%)		11 (12%)	
	80th centile	99 (55.9%)	71 (60.2%)		53 (57.6%)	
	100th centile	15 (8.5%)	26 (22%)		28 (30.4%)	
Kidney prognosis	1st Quintile	25 (14.1%)	15 (12.7%)	0.28	5 (5.4%)	0.0007
	2nd Quintile	64 (36.2%)	36 (30.5%)		26 (28.3%)	
	3rd Quintile	57 (32.2%)	34 (28.8%)		25 (27.2%)	
	4th Quintile	21 (11.9%)	25 (21.2%)		30 (32.6%)	
	5th Quintile	10 (5.6%)	8 (6.8%)		6 (6.5%)	
Body fat percentage	1st Quintile	68 (38.4%)	33 (28.7%)	0.002	33 (35.9%)	0.19
	2nd Quintile	73 (41.2%)	37 (32.1%)		34 (37%)	
	3rd Quintile	26 (14.7%)	34 (29.6%)		16 (17.4%)	
	4th Quintile	10 (5.6%)	7 (6.1%)		6 (6.5%)	
	5th Quintile	0 (0%)	4 (3.5%)		3 (3.2%)	
Lean body mass	1st Quintile	14 (7.9%)	13 (11.3%)	0.3	10 (10.9%)	0.6
	2nd Quintile	34 (19.2%)	29 (25.2%)		18 (19.6%)	
	3rd Quintile	51 (28.8%)	32 (27.8%)		28 (30.4%)	
	4th Quintile	66 (37.3%)	31 (27%)		27 (29.3%)	
	5th Quintile	12 (6.8%)	10 (8.7%)		9 (9.8%)	

Maternal plasma samples from discovery cohort (Melbourne, Australia; FLAG and BUMPS cohorts) were collected at 36 weeks' gestation, before FGR, or preeclampsia diagnosis. Data presented as number (%) in each risk bin. FGR defined as birthweight <3rd centile. Fishers exact test used for categorical variables. p-value <0.05 is considered significant. Note: Visceral Fat 40th centile data is not available.

Regarding metabolic parameters, SomaSignal Tests among women who subsequently delivered infants with fetal growth restriction (compared with control pregnancies) were strongly associated with increased visceral fat (median 434 control vs. 547.5 FGR; p = 4.06 × 10^−6^, Mann Whitney U test, Supplementary Figure S1a; unadjusted and adjusted median differences presented in Supplementary Table S3), with a 2.59-fold increase in patients classified in the 80th–100th centile category (22% FGR vs. 8.5% control, p = 0.0003, Fisher's exact test, Table 1); and body fat percentage (median 32 control vs. 34 FGR; p = 0.003, Mann Whitney U test, Supplementary Figure S1b; unadjusted and adjusted median differences presented in Supplementary Table S3), with an overall shift in higher body fat percentage quintiles (3.5% FGR in the 5th Quintile, compared to 0% controls, p = 0.002, Fisher's exact test, Table 1). Lean body mass (Supplementary Figure S1c), glucose tolerance (Supplementary Figure S1d), kidney prognosis (Supplementary Figure S1e), liver fat (Supplementary Figure S1f), alcohol impact (Supplementary Figure S1g), resting energy rate (Supplementary Figure 1h), non-alcoholic steatohepatitis ballooning (NASH–hepatocellular ballooning, Supplementary Figure 2a) and non-alcoholic steatohepatitis fibrosis (NASH, Supplementary Figure S2b) had similar risk profiles between groups (categorical models shown in Table 2; unadjusted and adjusted median differences presented in Supplementary Table S3).

**Table 2:** SomaSignal test thresholds for categorical models.

SomaSignal test	Category models	Control (n = 177) (%)	Fetal growth restriction (n = 118) (%)	p-value	Preeclampsia (n = 92) (%)	p-value
Liver fat	No excess liver fat (<0.5)	176 (99.4%)	111 (96.5%)	0.08	89 (96.7%)	0.1
	Some excess liver fat (>0.5)	1 (0.6%)	4 (3.5%)		3 (3.3%)	
Glucose tolerance	Normal tolerance (<0.5)	0 (0%)	0 (0%)	>0.99	0 (0%)	>0.99
	Impaired tolerance (>0.5)	177 (100%)	118 (100%)		92 (100%)	
Alcohol impact	Low impact (<0.5)	177 (100%)	114 (99.1%)	0.4	92 (100%)	>0.99
	High impact (>0.5)	0 (0%)	1 (0.9%)		0 (0%)	
NASH ballooning	Healthy (<0.5)	170 (96%)	107 (93%)	0.3	79 (85.9%)	0.005
	At Risk (>0.5)	7 (4%)	8 (7%)		13 (14.1%)	
NASH fibrosis	Healthy (<0.5)	164 (92.7%)	107 (93%)	>0.99	78 (84.8%)	0.05
	At Risk (>0.5)	13 (7.3%)	8 (7%)		14 (15.2%)	

Maternal plasma samples from discovery cohort (Melbourne, Australia; FLAG and BUMPS cohorts) were collected at 36 weeks' gestation, before diagnosis of either FGR, or preeclampsia, compared to control. Data presented as number (%) in each risk bin. FGR defined as birthweight <3rd centile. Fishers exact test used for categorical variables. p-value is considered significant <0.05.

### Replication study (aim 2)

Our secondary aim was to determine whether similar patterns in the Australian cohort would be replicated in the independent POP cohort, collected from the UK. In the POPs cohort those who delivered with FGR, had increased smokers during pregnancy (p = 0.0009, Fisher's exact test), reduced birth weight (p < 0.0001, Mann Whitney U test) and birthweight centile (p < 0.0001, Mann Whitney U test, Supplementary Table S4). Maternal age, maternal BMI, GDM status, preeclampsia status, type of labour, mode of delivery, gestation at delivery and fetal sex had similar risk profiles (Supplementary Table S4).

The same 18 SomaSignal Tests were measured in the replication cohort collected from Cambridge, UK. Like the Melbourne cohort, SomaSignal Tests were associated with altered cardiovascular parameters in those later delivering a growth restricted infant (compared with unaffected pregnancies) including, 1) increased primary cardiovascular risk (p = 0.02, Mann Whitney U test, Fig. 1i; unadjusted and adjusted median differences presented in Supplementary Table S5), 2) reduced cardiorespiratory fitness (VO_2_, median 22.4 control vs. 21.35 FGR; p = 0.02, Mann Whitney U test, Fig. 1k; unadjusted and adjusted median differences presented in Supplementary Table S5), 3) increased risk of heart failure with reduced ejection fraction (HFrEF) within 6 months (median 1.15 control vs. 1.4 FGR; p = 2.16 × 10^−5^, Mann Whitney U test, Fig. 1l; unadjusted and adjusted median differences presented in Supplementary Table S5) and 12 months (median 2.0 control vs. 2.5 FGR; p = 1.93 × 10^−5^, Mann Whitney U test, Fig. 1m; unadjusted and adjusted median differences presented in Supplementary Table S5). SomaSignal Tests were also associated with increased mid-life dementia risk within 20 years (median 0.15 control vs. 0.21 FGR; p = 1.86 × 10^−5^, Mann Whitney U test, Fig. 1p; unadjusted and adjusted median differences presented in Supplementary Table S5) in those who birthed a growth restricted infant. There was a 1.98-fold increase in patients classified in the high-risk category for dementia (43.8% FGR vs. 22.1% cohort, p = 0.0006, Fisher's exact test, Table 3).

**Table 3:** SomaSignal test survival model risk bins.

SomaSignal test	Risk bins for survival models	Control (n = 172) (%)	Fetal growth restriction (n = 80) (%)	p-value
Primary cardiovascular risk	Low	171 (99.4%)	80 (100%)	>0.99
	Medium-Low	1 (0.6%)	0 (0%)	
	Medium-High	0 (0%)	0 (0%)	
	High	0 (0%)	0 (0%)	
Residual cardiovascular risk	Low	33 (19.2%)	19 (23.8%)	0.7
	Medium -Low	137 (79.7%)	60 (75%)	
	Medium-High	2 (1.1%)	1 (1.2%)	
	High	0 (0%)	0 (0%)	
Cardiorespiratory fitness	Low	166 (96.5%)	76 (95%)	0.7
	Fair	6 (3.5%)	4 (5%)	
	Good	0 (0%)	0 (0%)	
	Excellent	0 (0%)	0 (0%)	
Dementia	Low	8 (4.7%)	1 (1.2%)	0.0006
	Medium-Low	80 (46.5%)	20 (25%)	
	Medium-High	46 (26.7%)	24 (30%)	
	High	38 (22.1%)	35 (43.8%)	
Visceral fat	20th centile	1 (0.6%)	0 (0%)	0.7
	60th centile	36 (20.9%)	14 (17.5%)	
	80th centile	110 (64%)	51 (63.8%)	
	100th centile	25 (14.5%)	15 (18.7%)	
Kidney prognosis	1st Quintile	17 (9.9%)	7 (8.8%)	0.75
	2nd Quintile	51 (29.7%)	18 (22.5%)	
	3rd Quintile	60 (34.9%)	30 (37.5%)	
	4th Quintile	32 (18.6%)	18 (22.5%)	
	5th Quintile	12 (6.9%)	7 (8.7%)	
Body fat percentage	1st Quintile	64 (37.2%)	12 (15%)	<0.0001
	2nd Quintile	69 (40.1%)	30 (37.5%)	
	3rd Quintile	31 (18.1%)	25 (31.3%)	
	4th Quintile	4 (2.3%)	10 (12.5%)	
	5th Quintile	4 (2.3%)	3 (3.7%)	
Lean body mass	1st Quintile	10 (5.8%)	2 (2.5%)	0.07
	2nd Quintile	41 (23.8%)	18 (22.5%)	
	3rd Quintile	57 (33.1%)	30 (37.5%)	
	4th Quintile	48 (27.9%)	29 (36.3%)	
	5th Quintile	16 (9.4%)	1 (1.2%)	

Maternal plasma samples from replication cohort (Cambridge, UK, POPS cohort) were collected at 36 weeks' gestation, before diagnosis of FGR, compared to control. Data presented as number (%) in each risk bin. FGR defined as birthweight <3rd centile. Fishers exact test used for categorical variables. p-value is considered significant <0.05. Note: Visceral Fat 40th centile data is not available.

SomaSignal Tests associated with elevated body fat percentage (median 32.8 control vs. 35.85 FGR; p = 1.67 × 10^−5^, Mann Whitney U test, Supplementary Figure S1j; unadjusted and adjusted median difference presented in Supplementary Table S5) were observed in women from the Cambridge cohort later delivering with fetal growth restriction. We observed a shift in their body fat percentage profiles, with a greater proportion of patients shifting to higher quintiles (p < 0.0001, Fisher's exact test, Table 3). Remaining health indicators: Residual cardiovascular disease risk (Fig. 1j), heart failure with preserved ejection fraction (HFpEF) within 6 months (Fig. 1n) and 12 months (Fig. 1o), visceral fat (Supplementary Figure S1i), lean body mass (Supplementary Figure S1k), glucose tolerance (Supplementary Figure S1l), kidney prognosis (Supplementary Figure S1m), liver fat (Supplementary Figure S1n), alcohol impact (Supplementary Figure S1o), resting energy rate (Supplementary Figure S1p), non-alcoholic steatohepatitis ballooning (NASH–hepatocellular ballooning, Supplementary Figure S2c) and non-alcoholic steatohepatitis fibrosis (NASH, Supplementary Figure S2d) were unchanged (categorical models shown in Tables 3 and 4; unadjusted and adjusted median differences presented in Supplementary Table S5).

**Table 4:** SomaSignal test thresholds for categorical models.

SomaSignal test	Category models	Control (n = 172) (%)	Fetal growth restriction (n = 80) (%)	p-value
Liver Fat	No excess liver fat (<0.5)	167 (97.1%)	78 (97.5%)	>0.99
	Some excess liver fat (>0.5)	5 (2.9%)	2 (2.5%)	
Glucose tolerance	Normal tolerance (<0.5)	0 (0%)	0 (0%)	>0.99
	Impaired tolerance (>0.5)	172 (100%)	80 (100%)	
Alcohol impact	Low impact (<0.5)	172 (100%)	80 (100%)	>0.99
	High impact (>0.5)	0 (0%)	0 (0%)	
NASH ballooning	Healthy (<0.5)	124 (72.1%)	52 (65%)	0.3
	At Risk (>0.5)	48 (27.9%)	28 (35%)	
NASH fibrosis	Healthy (<0.5)	156 (90.7%)	79 (98.8%)	0.01
	At Risk (>0.5)	16 (9.3%)	1 (1.2%)	

Maternal plasma samples from replication cohort (Cambridge, UK, POPS cohort) were collected at 36 weeks' gestation, before diagnosis of FGR, compared to control. Data presented as number (%) in each risk bin. FGR defined as birthweight <3rd centile. Fishers exact test used for categorical variables. p-value is considered significant <0.05.

### Preeclampsia sample outcomes

We next assessed protein signatures in 92 samples from women who developed preeclampsia, compared with 177 who did not (Australian cohort).

SomaSignal Tests in those developing preeclampsia at term were associated with, 1) increased risk of residual cardiovascular event (median 12.7 control vs. 15.85 preeclampsia; p = 5.54 × 10^−6^, Mann Whitney U test, Fig. 2b; unadjusted and adjusted median differences presented in Supplementary Table S6); 2) reduced cardiorespiratory fitness (VO_2_, median 21.3 control vs. 20.55 preeclampsia; p = 0.0003, Mann Whitney U test, Fig. 2c; unadjusted and adjusted median differences presented in Supplementary Table S6); 3) increased risk of heart failure with reduced ejection fraction (HFrEF) within 6 months (median 1.2 control vs. 1.5 preeclampsia; p = 0.002, Mann Whitney U test, Fig. 2d; unadjusted and adjusted median differences presented in Supplementary Table S6) and 12 months (median 2.1 control vs. 2.6 preeclampsia; p = 0.002, Mann Whitney U test, Fig. 2e; unadjusted and adjusted median differences presented in Supplementary Table S6); 4) increased risk of heart failure with preserved ejection fraction (HFpEF) within 6 months (median 1.4 control vs. 1.5 preeclampsia; p = 0.001, Mann Whitney U test, Fig. 2f; unadjusted and adjusted median differences presented in Supplementary Table S6) and 12 months (median 2.5 control vs. 2.7 preeclampsia; p = 0.001, Mann Whitney U test, Fig. 2g; unadjusted and adjusted median differences presented in Supplementary Table S6).

**Fig. 2:**
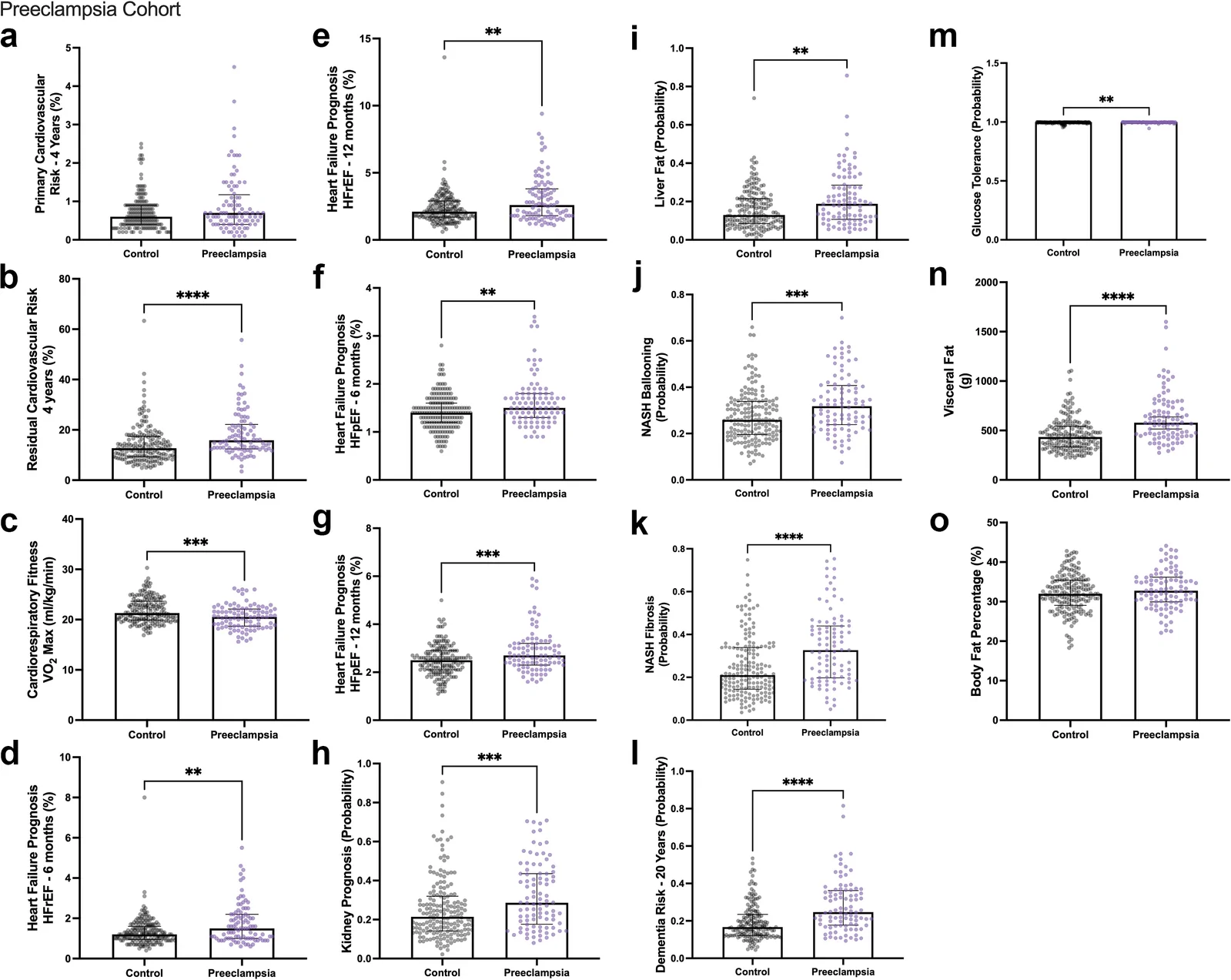
Bar graphs with median interquartile range (IQR) of SomaSignal Tests in plasma collected from a cohort collected from a hospital in Melbourne, Australia. Plasma was collected from pregnant women at 36 weeks' gestation, who developed preeclampsia (PE) at term, compared to those who did not (Control, n = 177, PE, n = 92). Risk of primary cardiovascular event (**a**), residual cardiovascular risk (**b**), cardiorespiratory fitness (VO_2_, **c**), heart failure with reduced ejection fraction (HFrEF) within 6 months (**d**), and 12 months (**e**), heart failure with preserved ejection fraction (HFpEF) within 6 months (**f**), and 12 months (**g**), risk of poor kidney prognosis (**h**), increased liver fat (**i**), non-alcoholic steatohepatitis ballooning (NASH–hepatocellular ballooning (**j**)), NASH fibrosis (**k**), risk of mid-life dementia (**l**), glucose intolerance (**m**), visceral fat (**n**) and body fat percentage (**o**). Groups compared using Mann–Whitney U tests with median ± IQR. ∗p < 0.05, ∗∗p < 0.01, ∗∗∗p < 0.001, ∗∗∗∗p < 0.0001.

Preeclampsia also had more pronounced SomaSignal Tests associated with future risk of kidney disease (median 0.214 control vs. 0.286 preeclampsia; p = 0.0007, Mann Whitney U test, Fig. 2h; unadjusted and adjusted median differences presented in Supplementary Table S6), with an increased shift towards higher kidney disease risk profiles (p = 0.0007, Fisher's exact test, Table 1). Liver fat (median 0.13 control vs. 0.188 preeclampsia; p = 0.001, Mann Whitney U test, Fig. 2i; unadjusted and adjusted median differences presented in Supplementary Table S6), non-alcoholic steatohepatitis (NASH) ballooning (median 0.259 control vs. 0.318 preeclampsia; p = 0.0005, Mann Whitney U test, Fig. 2j; unadjusted and adjusted median differences presented in Supplementary Table S6). Increased risk of NASH fibrosis (median 0.21 control vs. 0.3265 preeclampsia; p = 2.65 × 10^−6^, Mann Whitney U test, Fig. 2k; unadjusted and adjusted median differences presented in Supplementary Table S6) and dementia risk (median 0.167 control vs. 0.247 preeclampsia; p = 1.61 × 10^−8^, Mann Whitney U test, Fig. 2l; unadjusted and adjusted median differences presented in Supplementary Table S6), with a 2-fold increase in patients in the high-risk category (57.6% preeclampsia vs. 28.8% control, p < 0.0001, Fisher's exact test, Table 1).

SomaSignal Tests associated with adverse future metabolic diseases were also increased in those later diagnosed with preeclampsia: glucose intolerance (median 0.997 control vs. 0.998 preeclampsia; p = 0.001, Mann Whitney U test, Fig. 2m; unadjusted and adjusted median differences presented in Supplementary Table S6) and visceral fat (median 434 control vs. 579 preeclampsia; p = 5.35 × 10^−10^, Mann Whitney U test, Fig. 2n; unadjusted and adjusted median differences presented in Supplementary Table S6). Primary cardiovascular risk (Fig. 2a), body fat percentage (Fig. 2o), resting energy rate (Supplementary Figure S3a), lean body mass (Supplementary Figure S3b) and alcohol impact (Supplementary Figure S3c) remain unchanged (categorical models shown in Tables 1 and 2; unadjusted and adjusted median differences presented in Supplementary Table S6).

## Discussion

We report that pregnancies that later developed FGR and preeclampsia exhibit significantly altered maternal plasma protein signatures that are associated with risk of future chronic disease. Our findings in two prospective cohorts are consistent with large-scale, retrospective epidemiological studies which have linked FGR and preeclampsia to increased risk of maternal cardiovascular disease,^8^^,^^40^ metabolic disease,^41^ and Alzheimer's^11^, ^12^, ^13^ disease later in life.

Our findings align with work by Thilaganathan^42^ and others,^40^^,^^43^ suggesting that susceptibility to cardiovascular disease may precede pregnancy, or manifest decades before clinical symptoms arise. The more pronounced protein signatures in preeclampsia compared to FGR align with epidemiological data showing stronger associations between preeclampsia and subsequent cardiovascular disease.^40^ Our data supports the stress test hypothesis–that pregnancy imposes substantial cardiovascular and metabolic demands, unmasking latent cardiovascular and metabolic vulnerabilities that usually remain clinically undetected until later in life. As both cohorts had altered cardiovascular protein profiles before diagnosis of pregnancy complications, this indicates that protein signatures associated with future disease risk in non-pregnant populations are already detectible before clinical diagnosis.

As our samples were collected prior to the onset of pregnancy pathology symptoms, our data cannot determine whether pregnancy complications cause persistent cardiovascular damage, or, both outcomes have common determining factors such as chronic inflammation or endothelial and metabolic dysfunction. The markers altered before diagnosis of a pregnancy complication may reflect similar biological pathways common in both pregnancy complications and chronic disease development.

SomaSignal outputs are composite algorithm-derived measures, rather than single protein concentrations. Therefore, the contribution of individual proteins to the observed association with disease outcomes cannot be determined through these SomaSignal algorithms. Whilst each SomaSignal Test represents a predefined clinical domain analysed as an independent hypothesis, some may share underlying biological pathways. Therefore, these results should be interpreted carefully and require independent validation in longitudinal pregnancy cohorts. We acknowledge that assessment of multiple SomaSignal domains increases the possibility of false positive findings, particularly as several domains may reflect overlapping biological pathways. However, our findings highlight consistent changes across both the Australian and United Kingdom cohorts, and these replicated findings are less likely to represent isolated statistical associations.

A limitation of our study is that the SomaSignal Tests were not applied to cohorts where we have details of the long-term chronic disease outcomes. Such studies would require carefully designed large-scale cohorts with long-term follow up. It is also noted that this is the first time these SomaSignal Tests have been applied in pregnancy. However, the links between our exploratory analysis and those from large epidemiological studies^9^^,^^10^^,^^21^^,^^40^^,^^41^ provides evidence of biological plausibility. Therefore, we demonstrate biological concordance between pregnancy-associated proteomic profiles and previously derived disease-associated SomaSignal signature. We also note that some SomaSignal-derived traits (visceral fat and body fat percentage) do not reflect some clinical characteristics (BMI). Future work should refine these models using pregnancy samples, which is likely to enhance model performance.

We note there were key differences between the discovery (BUMPs/FLAG) and replication (POP) cohorts regarding clinical characteristics (such as maternal age, parity, gestational diabetes mellitus status, growth chart definitions and hospital-specific demographics). We were unable to control for this in the standard SomaSignal analysis. However, the findings from the two cohorts’ SomaSignal Tests were consistent. While established clinical characteristics such as gestational diabetes, BMI, and age are predictors of future chronic disease risk, SomaSignal Tests may capture additional biological information that is not reflected by these risk factors alone. Therefore, the clinical application could be to use SomaSignal results in combination with conventional risk factors, to support risk stratification and prevention strategies. This should be considered in future work where the protein signatures are modelled in pregnancy, and the heterogeneity of cohorts should be considered.

A considerable problem in the field of FGR research is the methods for determining ‘small’. In this study, we chose to utilise the same calculator (GROW) and applied population specific metrics to account for population differences across collection sites. We note a significant difference in parity between cohorts—with the Cambridge cohort only including nulliparous women. While there is strong evidence that FGR and preeclampsia are more common in first pregnancies,^44^^,^^45^ the fact that the SomaSignal tests perform similarly irrespective of this difference, and other differences in maternal characteristics between the two cohorts, suggests limited impact of these characteristics on test performance. However, as indicated earlier, this could be refined if the tests were trained specifically for pregnancy.

Although this study utilised a research-grade, multi-analyte platform, our findings provide proof-of-concept data that circulating proteomic signatures during pregnancy may reflect biological pathways linked with chronic disease risk later in life. This may be improved through model refinement or addition of clinical variables. Translation of these findings into clinical practice would require development of simplified, cost-effective assays, validation in independent cohorts, accessibility consideration, and smooth integration within existing pathways.

Pregnancy is a time when much of the women of reproductive age seek clinical care and undergo routine blood testing. It is a time that has been leveraged for opportunistic screening for other health conditions outside of the pregnancy (such as cervical cancer risk^46^). Our findings suggest it could also serve as a platform for assessing chronic disease risk. Early identification of high-risk women during pregnancy could enable targeted counselling, lifestyle intervention^47^ and structured postpartum surveillance^48^ to reduce the burden of future chronic disease.

Overall, our study has detected protein signatures in maternal plasma associated with pregnancy complications, and SomaSignal Tests associated with long-term chronic disease. Our findings suggest pregnancy may provide a potential window to identify women at increased long-term health risk. However, these exploratory findings require validation in large longitudinal cohorts before clinical application.

## Contributors

L.A.B.; Study design, data analysis, and wrote the first draft. S.P.W.; Study design, recruitment for the study and manuscript writing. T.M.M.; recruitment of patients, patient clinical characterisation and manuscript writing. D.I.; patient clinical characterisation and manuscript writing. P.C., T.V.N.; Performed laboratory studies, aliquoted the transfer of samples and manuscript writing. D.S.C.J., G.S.; Study design, recruitment of patients and manuscript writing. S.T., T.K.L.; Study design, data analysis, overall supervision of the study and manuscript writing. L.A.B and T.K.L both accessed and verified. All authors have read and approved the final version of the manuscript.

## Data sharing statement

Data generated and analysed during the current study are not publicly available at this time due to ongoing analyses of related biomarker datasets. However, once this is completed, data will be made available from the corresponding author upon reasonable request and investigator support. Correspondence and requests for materials should be addressed to L.A.B or T.J.K.L. Statistical analyses were performed using standard statistical software and packages. The packages and versions used have been described in the methods section.

## Declaration of interests

LAB declares one grant, and is named on one patent; DSCJ declares grants (Wellcome Leap, Roche Diagnostics, MRC, MRC), relationships with Medicines360, GondolaBio, Pfizer Inc and named on four patents; GS declares grants (Wellcome Leap, Roche Diagnostics Ltd, MRC), relationships with Natera Inc, Medicines360, Exeltis, Pfizer, named on 4 patents, and advisory board for Moderna, NGM Biopharmaceuticals Inc; SPW declares relationships with Diamedica Therapeutics and is named on 2 patents; ST declares relationships with Diamedica Therapeutics, Avilar Therapeutics, GondolaBio, Eli Lilly, Beech Biopharma and is named on 3 patents; TMM declares an NHMRC fellowship, named on 2 patents and is an examiner for RANZCOG; TKL declares ARC fellowship and is named on 3 patents. All other authors have no conflicts of interest to declare.
