# Supplementary material for: Proteomic signatures during pregnancy: an exploratory cohort study of long-term health indicators
Source: eBioMedicine. 2026 Sep 22;132:106491. doi: 10.1016/j.ebiom.2026.106491 (PMC13638798; doi:10.1016/j.ebiom.2026.106491)
Supplement: Supplementary file 1 [file mmc1.docx]

***Supplementary Table 1:*** *SomaSignal test information including name of test, number of features included, name of proteins (if disclosed), and method for model development.*

| SomaSignal Test | Number of features included | Name of proteins | Potential role in biology | Method for SomaSignal test development / Reference |
| --- | --- | --- | --- | --- |
| Primary cardiovascular risk – 4 years. | 13 proteins (13)  + Age  + Interaction term for each protein by Age (13 terms) | Top three proteins in the model disclosed:  -Gelsolin,  -Antithrombin III,  -sTREM-1 | -Gelsolin: Regulate cell morphology, differentiation, apoptosis, actin filament organisation; overexpression identified in cardiac hypertrophy  -Antithrombin III: may play a role in atherosclerosis.  -sTREM-1: increased levels associated with cardiovascular disease and associated organ dysfunction. | AFT-Weibull model with elastic net regularization  Reference: Williams, S. A., Kivimaki, M., Langenberg, C., Hingorani, A. D., Casas, J. P., Bouchard, C., ... & Wareham, N. J. (2019). Plasma protein patterns as comprehensive indicators of health. *Nature medicine*, *25*(12), 1851-1857. |
| RCV (Residual Cardiovascular; used in CVD-T2D SomaSignal) | 27 | Cartilage intermediate layer protein 2; Growth/differentiation factor 11/8; Spondin−1; Receptor−type tyrosine−protein phosphatase eta; Receptor tyrosine−protein kinase erbB−3; A disintegrin and metalloproteinase with thrombospondin motifs 13; NAD−dependent protein deacetylase sirtuin−2; Atrial natriuretic factor; Sushi, von Willebrand factor type A, EGF and pentraxin domain−containing protein 1; Protein phosphatase 1 regulator y subunit 1A; Triggering receptor expressed on myeloid cells 1; Protein kinase C−binding protein NELL1; Urokinase plasminogen activator surface receptor; Macrophage metalloelastase; Junctional adhesion molecule B; Anthrax toxin receptor 2; Natriuretic peptides B; Bifunctional heparan sulfate N−deacetylase/N−sulfotransferase 1; Trefoil factor 3; Immunoglobulin superfamily DCC subclass member 4; Golgi membrane protein 1; Inter−alpha−trypsin inhibitor heavy chain H2; Voltage−dependent calcium channel sub unit alpha−2/delta−3; Neural cell adhesion molecule 1, 120 kDa isoform; Low−density lipoprotein receptor−related protein 11; ADP−ribosylation factor−like protein 11; Mucin−16 (list as provided in Paterson et al., 27-protein RCV model) () | All 27 proteins and their association to cardiovascular disease is listed in supplementary material A in Williams et al. (2022). | AFT-Weibull model with LASSO regularization  Reference:  Williams, Stephen A., et al. "A proteomic surrogate for cardiovascular outcomes that is sensitive to multiple mechanisms of change in risk." Science translational medicine 14.639 (2022): eabj9625.  Paterson, C., Hagar, Y., Hinterberg, M. A., Charney, A. W., Valle, D. M. D., Filbin, M. R., ... & Williams, S. A. (2021). Application of a 27-protein candidate cardiovascular surrogate endpoint to track risk ascendancy and resolution in COVID-19. *medRxiv*, 2021-01. |
| Cardiorespiratory Fitness | 52 | Top three proteins in the model disclosed:  -Leptin  - C1QR1  -GGH | -Leptin: Produced in high amounts with BMI.  - C1QR1: Plays a role in the innate immune system.  -GGH: Requires folate – energy production and the repair of muscle tissue. | Regression model with elastic net regularization  Reference: Williams, S. A., Kivimaki, M., Langenberg, C., Hingorani, A. D., Casas, J. P., Bouchard, C., ... & Wareham, N. J. (2019). Plasma protein patterns as comprehensive indicators of health. *Nature medicine*, *25*(12), 1851-1857. |
| Heart failure prognosis (HFrEF) – 6 months | 17 | Not disclosed |  | AFT-Weibull model |
| Heart failure prognosis (HFrEF) – 12 months | 17 | Not disclosed |  | AFT-Weibull model |
| Heart failure prognosis (HFpEF) – 6 months | 14 | Not disclosed |  | AFT-Weibull model |
| Heart failure prognosis (HFpEF) – 12 months | 14 | Not disclosed |  | AFT-Weibull model |
| SomaSignal NASH: Steatosis  Note this model is only validated for serum. | 12 | Proteins included in the model:  - PTGR1  - GUSB  - INHBC  - HEXB  - RECQL  -BPIFB1  - GH2  - INSL5  -FABP12  - ERN1  - GRID2  -CNDP1 | For full description of each proteins role in relation to NASH/NAFLD see supplementary MMC1 from Sanyal et al. 2023  - PTGR1: important in fatty acid metabolism.  - GUSB: breakdown of large sugar mlecules, recycling cellular waste.  - INHBC: important for growth, cell differentiation, development.  - HEXB: breakdown fats and sugars; important for optimal health of nerves.  - RECQL: important for DNA quality.  -BPIFB1: role in innate immunity.  - GH2: important in growth control.  - INSL5: gut hormone; roles in metabolism.  -FABP12: role in lipid metabolism.  - ERN1: role in unfolded protein response in the endoplasmic reticulum.  - GRID2: unknown function  -CNDP1: role in metabolism, oxidative stress and inflammation. | Logistic regression model with elastic net regularization   - Sanyal, A. J., Williams, S. A., Lavine, J. E., Neuschwander-Tetri, B. A., Alexander, L., Ostroff, R., ... & Tonascia, J. (2023). Defining the serum proteomic signature of hepatic steatosis, inflammation, ballooning and fibrosis in non-alcoholic fatty liver disease. *Journal of hepatology*, *78*(4), 693-703. - Vali, Y., Lee, J., Boursier, J., Petta, S., Wonders, K., Tiniakos, D., ... & Bossuyt, P. M. Comparative diagnostic accuracy of blood-based biomarkers for staging at-risk NASH in NAFLD: first results of the LITMUS project. Research synthesis, translation and implementation of non-invasive liver tests, 75. - Brown, E. A., Minnich, A., Sanyal, A. J., Loomba, R., Du, S., Schwarz, J., ... & Charles, E. D. (2023). Effect of pegbelfermin on NASH and fibrosis-related biomarkers and correlation with histological response in the FALCON 1 trial. JHEP Reports, 5(4), 100661. |
| SomaSignal NASH: Inflammation  Note this model was only validated for serum | 14 | Proteins included in the model:  - ACY1  - TXNRD1  - FCGR3B  - PCOLCE2  - ADIPOQ  - RPN1  - GSTZ1  - PYY  - CCL23  - ACP1  - CTCF  - SAA2  - C1orf198  - TACSTD2 | For full description of each proteins role in relation to NASH/NAFLD see supplementary MMC1 from Sanyal et al. 2023  -ACY1: maintaining balance in metabolism; liver disease biomarker  - TXNRD1: antioxidant balance.  - FCGR3B: immune activation molecule.  - PCOLCE2: plays a role in tissue structure.  - ADIPOQ: adipose tissue metabolism.  - RPN1: involved in rough endoplasmic reticulum.  - GSTZ1: amino acid metabolism.  - PYY: plays a role in glucose metabolism and satiety.  - CCL23: immune cell molecule.  - ACP1: cell signalling, cell growth.  - CTCF: role in epigenetic regulation  - SAA2: responds to tissue injury and inflammation.  - C1orf198: unknown biological functions  - TACSTD2: cell surface signalling, cell proliferation, survival. | Logistic regression model with elastic net regularization   - Sanyal, A. J., Williams, S. A., Lavine, J. E., Neuschwander-Tetri, B. A., Alexander, L., Ostroff, R., ... & Tonascia, J. (2023). Defining the serum proteomic signature of hepatic steatosis, inflammation, ballooning and fibrosis in non-alcoholic fatty liver disease. *Journal of hepatology*, *78*(4), 693-703. - Vali, Y., Lee, J., Boursier, J., Petta, S., Wonders, K., Tiniakos, D., ... & Bossuyt, P. M. Comparative diagnostic accuracy of blood-based biomarkers for staging at-risk NASH in NAFLD: first results of the LITMUS project. Research synthesis, translation and implementation of non-invasive liver tests, 75. - Brown, E. A., Minnich, A., Sanyal, A. J., Loomba, R., Du, S., Schwarz, J., ... & Charles, E. D. (2023). Effect of pegbelfermin on NASH and fibrosis-related biomarkers and correlation with histological response in the FALCON 1 trial. JHEP Reports, 5(4), 100661. |
| SomaSignal NASH: Ballooning | 5 | Proteins included in the model:  - AKR1B10  - PTGR1  - ADAMTSL2  - CNN2  - CTLA4 | For full description of each proteins role in relation to NASH/NAFLD see supplementary MMC1 from Sanyal et al. 2023  - AKR1B10: plays a role in protection from oxidative stress.  - PTGR1: plays a role in inflammation.  - ADAMTSL2: regulating cell growth and differentiation.  - CNN2: plays a role in cell migration, adhesion and proliferation.  - CTLA4: immune molecule. | Logistic regression model with elastic net regularization   - Sanyal, A. J., Williams, S. A., Lavine, J. E., Neuschwander-Tetri, B. A., Alexander, L., Ostroff, R., ... & Tonascia, J. (2023). Defining the serum proteomic signature of hepatic steatosis, inflammation, ballooning and fibrosis in non-alcoholic fatty liver disease. *Journal of hepatology*, *78*(4), 693-703. - Vali, Y., Lee, J., Boursier, J., Petta, S., Wonders, K., Tiniakos, D., ... & Bossuyt, P. M. Comparative diagnostic accuracy of blood-based biomarkers for staging at-risk NASH in NAFLD: first results of the LITMUS project. Research synthesis, translation and implementation of non-invasive liver tests, 75. - Brown, E. A., Minnich, A., Sanyal, A. J., Loomba, R., Du, S., Schwarz, J., ... & Charles, E. D. (2023). Effect of pegbelfermin on NASH and fibrosis-related biomarkers and correlation with histological response in the FALCON 1 trial. JHEP Reports, 5(4), 100661. |
| SomaSignal NASH: Fibrosis | 8 | Proteins included in the model:  - ADAMTSL2  - COLEC11  - NFASC  - C7  - FCRL3  - KDR  - PLOD3  - WNT5A | For full description of each proteins role in relation to NASH/NAFLD see supplementary MMC1 from Sanyal et al. 2023  - ADAMTSL2: regulating cell growth and differentiation.  - COLEC11: plays a role in embryonic development and innate immunity.  - NFASC: involved in nervous system cellular structure and function.  - C7: innate immune system.  - FCRL3: inflammation.  - KDR: important for angiogenesis, survival and migration.  - PLOD3: important for tissue structure.  - WNT5A: important for cell migration, proliferation pathways. | Logistic regression model with elastic net regularization   - Sanyal, A. J., Williams, S. A., Lavine, J. E., Neuschwander-Tetri, B. A., Alexander, L., Ostroff, R., ... & Tonascia, J. (2023). Defining the serum proteomic signature of hepatic steatosis, inflammation, ballooning and fibrosis in non-alcoholic fatty liver disease. *Journal of hepatology*, *78*(4), 693-703. - Vali, Y., Lee, J., Boursier, J., Petta, S., Wonders, K., Tiniakos, D., ... & Bossuyt, P. M. Comparative diagnostic accuracy of blood-based biomarkers for staging at-risk NASH in NAFLD: first results of the LITMUS project. Research synthesis, translation and implementation of non-invasive liver tests, 75. - Brown, E. A., Minnich, A., Sanyal, A. J., Loomba, R., Du, S., Schwarz, J., ... & Charles, E. D. (2023). Effect of pegbelfermin on NASH and fibrosis-related biomarkers and correlation with histological response in the FALCON 1 trial. JHEP Reports, 5(4), 100661. |
| Dementia SomaSignal Test (dSST) | Model based (protein signature)  25 proteins | Full list available in sTable 4 Duggan et al 2025  Top 3 ranked by effect size:  Pleitrophin  Neuronal pentraxin receptor  Tubulin-specific chaperone A |  | AFT-Weibull model with LASSO regularization   - Duggan, M. R., Paterson, C., Lu, Y., Biegel, H., Dark, H. E., Cordon, J., ... & Walker, K. A. (2025). The Dementia SomaSignal Test (dSST): A plasma proteomic predictor of 20‐year dementia risk. Alzheimer's & Dementia, 21(2), e14549. |
| Glucose tolerance | 41 | Not disclosed. | - | - Elastic net regression model |
| Visceral Fat | 51 | Top three proteins in the model disclosed:  - Leptin  - FABPA  - INHBC | - Leptin: Produced in high amounts with BMI.  - FABPA: Linked to metabolic and immation.  - INHBC: Plays a role in boy composition and energy expenditure. | - Elastic net linear regression model   Reference: Williams, S. A., Kivimaki, M., Langenberg, C., Hingorani, A. D., Casas, J. P., Bouchard, C., ... & Wareham, N. J. (2019). Plasma protein patterns as comprehensive indicators of health. *Nature medicine*, *25*(12), 1851-1857. |
| Body fat percentage | 219 | Top three proteins in the model disclosed:  - Leptin  - FABP (fatty acid-binding protein)  - SFRP4 (secreted frizzled-related  protein 4)  Full list of proteins available in Supplemental table 1 Williams et al. 2019 | - Leptin: Produced in high amounts with BMI.  - FABP (fatty acid-binding protein): Fatty acid metabolism.  - SFRP4 (secreted frizzled-related  protein 4): roles in adipose tissue in obesity. | - Elastic net regression model   Reference: Williams, S. A., Kivimaki, M., Langenberg, C., Hingorani, A. D., Casas, J. P., Bouchard, C., ... & Wareham, N. J. (2019). Plasma protein patterns as comprehensive indicators of health. *Nature medicine*, *25*(12), 1851-1857. |
| Resting energy rate | 122 | Top three proteins in the model disclosed:  - Leptin  - IGLO5 (IgLON family member 5)  - ATF6A (cyclic AMP-dependent  transcription factor ATF-6 alpha) | - Leptin: Produced in high amounts with BMI.  - IGLO5 (IgLON family member 5): no known biological function  - ATF6A (cyclic AMP-dependent  transcription factor ATF-6 alpha): involved in unfolded protein response during endoplasmic reticulum stress. | - Elastic net linear regression model   Reference: Williams, S. A., Kivimaki, M., Langenberg, C., Hingorani, A. D., Casas, J. P., Bouchard, C., ... & Wareham, N. J. (2019). Plasma protein patterns as comprehensive indicators of health. *Nature medicine*, *25*(12), 1851-1857. |
| Lean body mass | 115 | Top three proteins in the model disclosed:  - SEZ6L:  - SLIK4 SLIT and NTRK protein-  like 4:  - WISP-2  Full list of proteins available in Supplemental table 1 Williams et al. 2019 | - SEZ6L: Associated cardiometabolic conditions.  - SLIK4 SLIT and NTRK protein-  like 4: function in neurite growth  - WISP-2: adipokine secreted in adiposity. | - Elastic net linear regression model   Reference: Williams, S. A., Kivimaki, M., Langenberg, C., Hingorani, A. D., Casas, J. P., Bouchard, C., ... & Wareham, N. J. (2019). Plasma protein patterns as comprehensive indicators of health. *Nature medicine*, *25*(12), 1851-1857. |
| Alcohol impact | 100 | Top three proteins in the model disclosed:  - SCUB1 (signal peptide, CUB and  EGF-like domain-containing  protein 1)  - SERC (phosphoserine  aminotransferase)  - SCF (stem cell factor) kit ligand | - SCUB1 (signal peptide, CUB and  EGF-like domain-containing  protein 1): Promotes platelet-to-platelet interaction.  - SERC (phosphoserine  aminotransferase): No known biological function.  - SCF (stem cell factor) kit ligand: Involved in haematopoiesis in adults. | - Elastic net logistic regression model   Reference: Williams, S. A., Kivimaki, M., Langenberg, C., Hingorani, A. D., Casas, J. P., Bouchard, C., ... & Wareham, N. J. (2019). Plasma protein patterns as comprehensive indicators of health. *Nature medicine*, *25*(12), 1851-1857. |
| Kidney Prognosis | 10 | Top three proteins in the model disclosed:  - TMEDA (transmembrane  emp24 domain-containing  protein 10)  - Apo A-IV (apolipoprotein A-IV)  -Beta-2 microglobulin | -TMEDA (transmembrane  emp24 domain-containing  protein 10): plays a role in kidney development.  - Apo A-IV (apolipoprotein A-IV): Associated with kidney disease; lipid binding protein  -Beta-2 microglobulin: Measure of kidney filtration | - Logistic regression model   Reference: Williams, S. A., Kivimaki, M., Langenberg, C., Hingorani, A. D., Casas, J. P., Bouchard, C., ... & Wareham, N. J. (2019). Plasma protein patterns as comprehensive indicators of health. *Nature medicine*, *25*(12), 1851-1857. |
| Liver Fat | 60 | Top three proteins in the model disclosed:  - SEZ6L (seizure 6-like protein)  - FABPA (fatty acid-binding  protein, adipocyte)  - IGFBP-1 (insulin-like growth  factor-binding protein 1) | - SEZ6L (seizure 6-like protein): associated with cardiometabolic conditions  - FABPA (fatty acid-binding  protein, adipocyte): associated with non-alcoholic liver disease.  - IGFBP-1 (insulin-like growth  factor-binding protein 1): Plays a role in metabolism and insulin resistance | - Elastic net logistic regression model   Reference: Williams, S. A., Kivimaki, M., Langenberg, C., Hingorani, A. D., Casas, J. P., Bouchard, C., ... & Wareham, N. J. (2019). Plasma protein patterns as comprehensive indicators of health. *Nature medicine*, *25*(12), 1851-1857. |

***Supplementary Table 2:*** *Discovery cohort - Maternal characteristics and pregnancy outcomes for FLAG and BUMPS (fetal growth restriction, FGR) and preeclampsia plasma samples collected at 36 weeks’ gestation. Data presented as mean (standard deviation) if normally distributed data, as median [25th – 75th percentile] if not normally distributed data, and as number (%) if categorical. FGR defined as birthweight <3rd centile. BMI = Body mass index. Mann-Whitney test was used for non-parametric data, and Fisher’s exact tests for categorical variables. p-value is considered significant <0.05.*

|  | **Control (n=177)** | **FGR (<3^rd^, n=118)** | **p-value** | **Preeclampsia (n=92)** | **p-value** |
| --- | --- | --- | --- | --- | --- |
| **Maternal Age** (years)  Median (IQR) | 32.00 [29.50 – 35.50] | 34.00 [31.00 – 36.00] | 0.03 | 33.00 [30.00 – 36.00] | 0.37 |
| **BMI** (kg/m^2^)  Median (IQR) | 24.70 [22.03 – 28.32] | 24.74 [22.30 – 29.36] | 0.48 | 26.00 [22.60 – 29.35] | 0.17 |
| **Parity** no. (%)   - 0 - 1 - ≥2 | - 86 (48.6%) - 62 (35.0%) - 29 (16.4%) | - 74 (62.7%) - 32 (27.1%) - 12 (10.2%) | 0.05 | - 66 (72.5%) - 15 (16.5%) - 10 (11%) | 0.0007 |
| Cigarette Smoking   - Non-smoker - Smoker | - 170 (96.0%) - 7 (4.0%) | - 110 (93.2%) - 8 (6.8%) | 0.28 | - 86 (94.5%)  - 5 (5.5%) | 0.56 |
| Gestational Diabetes Mellitus   - No GDM - GDM – Diet Controlled - GDM – Insulin Controlled - Not listed | - 142 (80.2%) - 21 (11.9%) - 13 (7.3%) - 1 (0.6%) | - 92 (78%) - 18 (15.25%) - 7 (6%) - 1 (0.84%) | 0.81 | - 71 (78%) - 17 (18.7%) - 3 (3.3%) - 0 (0%) | 0.24 |
| Onset of labour   - Spontaneous - Induced - Augmentation of Labour - No labour | - 60 (34%) - 70 (39.5%) - 11 (6.2%) - 36 (20.3%) | - 23 (19.5%) - 57 (48.3%) - 5 (4.2%) - 33 (28%) | 0.03 | - 17 (18.7%) - 54 (59.3%) - 3 (3.3%) - 17 (18.7%) | 0.01 |
| Mode of Birth   - Vaginal birth - Instrumental delivery - Emergency Caesarean section - Caesarean section | - 78 (44.1%) - 29 (16.4%) - 39 (22.0%) - 31 (17.5%) | - 45 (38.1%) - 17 (14.4%) - 32 (27.1%) - 24 (20.4%) | 0.6 | - 29 (31.9%) - 11 (12.1%) - 38 (41.75%) - 13 (14.25%) | 0.009 |
| **Gestation at Delivery** (weeks)  Median (IQR) | 39.29 [38.71 – 40.22] | 38.8 [37.7 – 39.85] | <0.0001 | 38.86 [38.00 – 39.71] | 0.0002 |
| **Birth weight** (g)  Median (IQR) | 3480 [3210 – 3780] | 2510 [2278 – 2663] | <0.0001 | 3270 [2840 – 3650] | 0.0004 |
| **Birthweight centile**  **Median (IQR)** | 48.30 [24.90 – 77.80] | 1.5 [0.77 – 2.00] | <0.0001 | 41.00 [12.8 – 71.5] | 0.03 |
| **Male** no. (%) | 98 (55.3%) | 63 (53.4%) | 0.74 | 46 (51%) | 0.45 |

***Supplementary Table 3:*** *Unadjusted and adjusted median difference and 95% confidence intervals (95% CI) of SomaSignal Tests in Discovery cohort - FLAG and BUMPS (fetal growth restriction, FGR, n=118, vs Control, n=177). All results are reported as median difference with 95% CI. Values were adjusted for maternal age, maternal body mass index (BMI), smoking status, and gestational diabetes mellitus status.*

| SomaSignal Tests | **Unadjusted median difference (95% CI)** | **Adjusted median difference (95% CI)** |
| --- | --- | --- |
| Alcohol Impact | -0.002 (-0.003 to 0.001) | -0.002 (-0.0046 to 0.0013) |
| Body fat percentage | 2.0 (0.7 to 3.0) | 1.74 (0.69 to 2.79) |
| Cardiorespiratory fitness (VO_2_) | -0.5 (-1.4 to -0.2) | -0.49 (-1.29 to 0.31) |
| Dementia | 0.1120 (0.061 to 0.12) | 0.10 (0.063 to 0.14) |
| Glucose tolerance | 0.0005 (0.000 to 0.001) | 0.0006 (-0.0003 to 0.0016) |
| Heart Failure with preserved Ejection Fraction (HFpEF) within 12 months | 0.0 (-0.1 to 0.2) | -0.006 (-0.16 to 0.15) |
| Heart Failure with preserved Ejection Fraction (HFpEF) within 6 months | 0.0 (-0.1 to 0.1) | 0.015 (-0.084 to 0.11) |
| Heart Failure with reduced Ejection Fraction (HFrEF) within 12 months | 0.5 (0.2 to 0.6) | 0.36 (-0.017 to 0.74) |
| Heart Failure with reduced Ejection Fraction (HFrEF) within 6 months | 0.3 (0.1 to 0.4) | 0.22 (0.0038 to 0.44) |
| Kidney prognosis | 0.042 (-0.005 to 0.063) | 0.034 (-0.017 to 0.084) |
| Lean body mass | -1.150 (-1.7 to 0.5) | -1.25 (-2.61 to -0.10) |
| Liver fat | 0.013 (-0.007 to 0.034) | -0.005 (-0.033 to 0.022) |
| Non-alcoholic steatohepatitis ballooning (NASH - hepatocellular ballooning) | 0.011 (-0.02 to 0.033) | 0.0036 (-0.039 to 0.046) |
| Non-alcoholic steatohepatitis fibrosis | 0.000 (-0.038 to 0.02) | 0.0046 (-0.04 to 0.049) |
| Primary cardiovascular risk | 0.2 (0.1 to 0.3) | 0.13 (0.032 to 0.23) |
| Residual cardiovascular risk | 1.0 (-0.3 to 2.3) | 0.77 (-0.75 to 2.30) |
| Resting energy rate | -15.00 (-85.00 to 24.00) | -61.01 (-116.86 to -5.16) |
| Visceral fat | 113.5 (56.00 to 137.0) | 83.64 (33.90 to 133.37) |

***Supplementary Table 4:*** *Replication cohort - Maternal characteristics and pregnancy outcomes for POP study (fetal growth restriction, FGR) plasma samples collected at 36 weeks’ gestation. Data presented as mean (standard deviation) if normally distributed data, as median [25th – 75th percentile] if not normally distributed data, and as number (%) if categorical. FGR defined as birthweight <3rd centile. BMI = Body mass index. Mann-Whitney test was used for non-parametric data, and Fisher’s exact tests for categorical variables. p-value is considered significant <0.05.*

|  | **Control (n=172)** | **FGR (<3^rd^, n=80)** | **p-value** |
| --- | --- | --- | --- |
| **Maternal Age** (years)  Median (IQR) | 30.50 [26.00 – 34.00] | 30.00 [27.00 – 34.00] | 0.81 |
| **BMI** (kg/m^2^)  Median (IQR) | 23.85 [21.98 – 27.49] | 24.55 [22.68 – 28.56] | 0.11 |
| Cigarette Smoking   - Non-smoker - Smoker | - 165 (95.9%) - 7 (4.1%) | - 66 (82.5%) - 14 (17.5%) | 0.0009 |
| Gestational Diabetes Mellitus   - No GDM - GDM – Diet Controlled - GDM – Insulin Controlled - Type 1 or Type 2 Diabetes | - 161 (93.6%) - 6 (3.5%) - 5 (2.9%) - 0 (0%) | - 78 (97.5%) - 0 (0%) - 1 (1.25%) - 1 (1.25%) | 0.12 |
| Preeclampsia | 11 (6.4%) | 8 (10%) | 0.32 |
| Onset of labour   - Spontaneous - Not spontaneous | - 99 (57.5%) - 73 (42.5%) | - 43 (53.75%) - 37 (46.25%) | 0.59 |
| Mode of Birth   - Vaginal birth - Instrumental delivery - Emergency Caesarean section - Caesarean section - Not listed | - 94 (54.6%) - 33 (19.2%) - 25 (14.5%) - 18 (10.5%) - 2 (1.2%) | - 43 (53.75%) - 14 (17.5%) - 9 (11.25%) - 14 (17.5%) - 0 (0%) | 0.47 |
| **Gestation at Delivery** (weeks)  Median (IQR) | 40.50 [39.14 – 41.14] | 40.00 [39.14 – 40.86] | 0.2 |
| **Birth weight** (g)  Median (IQR) | 3425 [3103 – 3720] | 2598 [2358 – 2758] | <0.0001 |
| **Birthweight centile**  **Median (IQR)** | 40.55 [20.28 – 66.13] | 1.35 [0.525 – 2.1] | <0.0001 |
| **Male** no. (%) | 90 (52.3%) | 42 (52.5%) | 0.98 |

***Supplementary Table 5:*** *Unadjusted and adjusted median difference and 95% confidence intervals (95% CI) of SomaSignal Tests in Replication cohort – POP, Cambridge, UK study (fetal growth restriction, FGR, n=80, vs Control, n=172) plasma samples collected at 36 weeks’ gestation. All results are reported as median difference with 95% CI. Values were adjusted for maternal age, maternal body mass index (BMI), smoking status, and gestational diabetes mellitus status.*

| SomaSignal Tests | **Unadjusted median difference (95% CI)** | **Adjusted median difference (95% CI)** |
| --- | --- | --- |
| Alcohol Impact | 0.000 (-0.002 to 0.002) | 0.0008 (-0.002 to 0.0039) |
| Body fat percentage | 3.05 (1.6 to 4.1) | 2.71 (1.26 to 4.17) |
| Cardiorespiratory fitness (VO_2_) | -1.050 (-1.4 to -0.10) | -0.45 (-1.17 to 0.27) |
| Dementia | 0.059 (0.03 to 0.078) | 0.055 (0.022 to 0.087) |
| Glucose tolerance | 0.000 (-0.001 to 0.000) | -0.0005 (-0.002 to 0.0009) |
| Heart Failure with preserved Ejection Fraction (HFpEF) within 12 months | 0.00 (-0.2 to 0.2) | 0.033 (-0.22 to 0.30) |
| Heart Failure with preserved Ejection Fraction (HFpEF) within 6 months | 0.00 (-0.1 to 0.1) | 0.038 (-0.088 to 0.16) |
| Heart Failure with reduced Ejection Fraction (HFrEF) within 12 months | 0.5 (0.3 to 0.8) | 0.46 (0.15 to 0.77) |
| Heart Failure with reduced Ejection Fraction (HFrEF) within 6 months | 0.25 (0.2 to 0.4) | 0.26 (0.07 to 0.44) |
| Kidney prognosis | 0.0355 (-0.008 to 0.07) | 0.034 (-0.02 to 0.08) |
| Lean body mass | 0.30 (-0.9 to 1.2) | -0.67 (-1.76 to 0.43) |
| Liver fat | 0.025 (-0.003 to 0.049) | 0.01 (-0.02 to 0.04) |
| Non-alcoholic steatohepatitis ballooning (NASH - hepatocellular ballooning) | 0.029 (-0.011 to 0.066) | 0.03 (-0.01 to 0.08) |
| Non-alcoholic steatohepatitis fibrosis | -0.012 (-0.052 to 0.012) | -0.0003 (-0.06 to 0.06) |
| Primary cardiovascular risk | 0.000 (0.00 to 0.10) | 0.048 (-0.045 to 0.14) |
| Residual cardiovascular risk | -0.25 (-1.6 to 0.5) | -0.59 (-1.96 to 0.79) |
| Resting energy rate | -28.50 (-72.00 to 38.00) | -52.58 (-118.25 to 13.08) |
| Visceral fat | 58.50 (-2.0 to 90.0) | 21.30 (-33.93 to 76.52) |

***Supplementary Table 6:*** *Unadjusted and adjusted median difference and 95% confidence intervals (95% CI) of SomaSignal Tests in Discovery cohort - FLAG and BUMPS (preeclampsia, PE, n=92, vs Control, n=177). All results are reported as median difference with 95% CI. Values were adjusted for maternal age, maternal body mass index (BMI), smoking status, and gestational diabetes mellitus status.*

| SomaSignal Tests | **Unadjusted median difference (95% CI)** | **Adjusted median difference (95% CI)** |
| --- | --- | --- |
| Alcohol Impact | -0.003 (-0.003 to 0.001) | -0.0018 (-0.004 to 0.0008) |
| Body fat percentage | 0.75 (-0.4 to 2.0) | -0.02 (-1.21 to 1.16) |
| Cardiorespiratory fitness (VO_2_) | -0.75 (-1.9 to -0.6) | -0.64 (-1.35 to 0.06) |
| Dementia | 0.08 (0.051 to 0.104) | 0.089 (0.048 to 0.13) |
| Glucose tolerance | 0.001 (0.000 to 0.002) | 0.0009 (0.000012 to 0.0019) |
| Heart Failure with preserved Ejection Fraction (HFpEF) within 12 months | 0.2 (0.1 to 0.5) | 0.21 (-0.017 to 0.43) |
| Heart Failure with preserved Ejection Fraction (HFpEF) within 6 months | 0.1 (0.1 to 0.3) | 0.12 (-0.004 to 0.24) |
| Heart Failure with reduced Ejection Fraction (HFrEF) within 12 months | 0.5 (0.1 to 0.7) | 0.41 (-0.11 to 0.94) |
| Heart Failure with reduced Ejection Fraction (HFrEF) within 6 months | 0.3 (0.1 to 0.4) | 0.24 ( -0.04 to 0.51) |
| Kidney prognosis | 0.072 (0.026 to 0.103) | 0.07 (0.0067 to 0.13) |
| Lean body mass | -0.25 (-1.4 to 0.8) | -0.05 (-1.47 to 1.37) |
| Liver fat | 0.058 (0.016 to 0.069) | 0.035 (0.005 to 0.066) |
| Non-alcoholic steatohepatitis ballooning (NASH - hepatocellular ballooning) | 0.059 (0.021 to 0.079) | 0.038 (-0.011 to 0.087) |
| Non-alcoholic steatohepatitis fibrosis | 0.117 (0.053 to 0.13) | 0.12 (0.058 to 0.17) |
| Primary cardiovascular risk | 0.1 (0.0 to 0.2) | 0.05 (-0.05 to 0.14) |
| Residual cardiovascular risk | 3.15 (2.2 to 5.1) | 3.71 (1.68 to 5.73) |
| Resting energy rate | 0.00 (-53.00 to 74.00) | -29.75 (-97.21 to 37.71) |
| Visceral fat | 145.0 (102.0 to 192.0) | 108.43 (48.74 to 168.13) |

*Supplementary figure 1*: Bar graphs with median interquartile range (IQR) of SomaSignal Tests in two large cohorts where plasma was collected from pregnant women at 36 weeks’ gestation, who subsequently delivered with fetal growth restriction (FGR), with birthweight <3^rd^ centile, or >3^rd^ birthweight centile (control) (**a-h**, Discovery cohort, **i-p**, Replication cohort). visceral fat (**a**), body fat percentage (**b**), lean body mass (**c**), glucose intolerance (**d**), risk of poor kidney prognosis (progressive chronic renal insufficiency, **e**), increased liver fat (**f**), alcohol impact (**g**) and resting energy rate (**h**) in the discovery cohort, collected at a hospital in Melbourne, Australia (Control, n=177, FGR, n=118). visceral fat (**i**), body fat percentage (**j**), lean body mass (**k**), glucose intolerance (**l**), risk of poor kidney prognosis (**m**), increased liver fat (**n**), alcohol impact (**o**) and resting energy rate (**p**) in the replication cohort, collected at a hospital in Cambridge, United Kingdom (Control, n=172, FGR, n=80). Groups compared using Mann-Whitney U tests with median ± IQR. **p*<0.05, ***p*<0.01, ****p*<0.001, **** *p*<0.0001.

*Supplementary figure 2:* Bar graphs with median interquartile range (IQR) of SomaSignal Tests in two large cohorts where plasma was collected from pregnant women at 36 weeks’ gestation, who subsequently delivered with fetal growth restriction (FGR), birthweight <3^rd^ centile, or birthweight >3^rd^ centile (control) (**a-b**, Discovery cohort, **c-d,** Replication cohort). Risk of non-alcoholic steatohepatitis ballooning (NASH - hepatocellular ballooning, **a**) and NASH fibrosis (**b**) in the discovery cohort, collected at a hospital in Melbourne, Australia (Control, n=177, FGR, n=118). Risk of NASH (hepatocellular ballooning, **c**) and NASH fibrosis (**d**) in the replication cohort, collected at a hospital in Cambridge, United Kingdom (Control, n=172, FGR, n=80). Groups compared using Mann-Whitney U tests with median ± IQR.

*Supplementary figure 3:* Bar graphs of SomaSignal Tests in plasma collected from a cohort collected from a hospital in Melbourne, Australia. Plasma was collected from pregnant women at 36 weeks’ gestation, who subsequently developed preeclampsia (PE) at term, compared to those who did not (Control, n=177, PE, n=92). Data shows resting energy rate (**a**), lean body mass (**b**), and alcohol impact (**c**). Groups compared using Mann-Whitney U tests with median ± IQR. **p*<0.05, ***p*<0.01, ****p*<0.001, **** *p*<0.0001.
